# Not the Typical Pneumonia: An Unusual Case of Rasburicase-induced Methomoglobinemia

**DOI:** 10.7759/cureus.3084

**Published:** 2018-08-01

**Authors:** Moustafa Younis, Laith Derbas, Stephen M Eikermann, Majdi S Hamarshi

**Affiliations:** 1 Internal Medicine, University of Missouri/St. Luke's Health System, Kansas City, USA; 2 Internal Medicine, University of Missouri, Kansas City, USA; 3 Critical Care, University of Missouri/St. Luke's Health System, Kansas City, USA; 4 Critical Care, St Luke's Health System, Kansas City, USA

**Keywords:** methemoglobinemia, rasburicase, hemolysis, glucose-6-phosphate dehydrogenase deficiency

## Abstract

We present a rare case of rasburicase-induced methemoglobinemia and hemolytic anemia in the setting of presumed glucose-6-phosphate dehydrogenase (G6PD) deficiency. A 78-year-old male with a known history of chronic lymphocytic leukemia presented to the clinic with fever of unknown origin. Laboratory results were significant for hyperuricemia. He was empirically started on levofloxacin and rasburicase. He then presented to the emergency department with shortness of breath and syncope. Physical examination was remarkable for a fever of 102.8 °F, conjunctival pallor, and scleral icterus. An infiltrate was observed on his computed tomography (CT) angiogram of the chest. Arterial blood gas on 50% fraction of inspired oxygen was significant for an arterial oxygen level of 222 millimeters mercury and oxyhemoglobin of 85.9%. Co-oximetry was then obtained and methemoglobin level was 13.4%. Laboratory results were noteworthy for a drop-in hemoglobin, indirect hyperbilirubinemia, low haptoglobin and elevated lactate dehydrogenase; depicting hemolytic anemia. The patient received two units of packed red blood cells, intravenous broad-spectrum antibiotics and he clinically improved.

## Introduction

Tumor lysis syndrome (TLS) is an oncological emergency referring to the constellation of metabolic disturbances resulting from the rapid destruction of tumor cells leading to the release of intracellular ions and nucleic acids, eventually metabolized to uric acid, into the blood stream [1]. The metabolic disturbances associated with TLS include hyperuricemia, hyperkalemia, hyperphosphatemia, hypocalcemia, and acute kidney injury (AKI) [2]. TLS can be precipitated either following the administration of cytotoxic chemotherapeutic agents or spontaneously in the setting of hematological malignancies [3].

In TLS, the main prophylactic methods include intravenous hydration, allopurinol and rasburicase administration [4]. For patients at high risk for TLS or in those where allopurinol is contraindicated, rasburicase is currently recommended by the Guidelines for the Management of Pediatric and Adult Tumor lysis syndrome published by the American Society of Clinical Oncology in 2008 [4]. Rasburicase is a recombinant urate-oxidase enzyme approved by the Food and Drug Administration (FDA) for the treatment and/or prevention of hyperuricemia and TLS. It converts uric acid to allantoin (an inactive and soluble metabolite of uric acid) facilitating the renal clearance of uric acid and decreasing its precipitation in the kidneys [5]. Multiple studies demonstrated a greater reduction in uric acid levels with a lower risk of AKI, compared to allopurinol [5-7].

Rasburicase is usually well tolerated; however, several side effects are of concern. Methemoglobinemia is an infrequent (<1%) but serious complication associated with the administration of rasburicase [8]. In addition, the drug is contraindicated in patients with glucose-6-phosphate dehydrogenase (G6PD) deficiency as hydrogen peroxide (a by-product of uric acid catabolism) can present an oxidative stress thus, precipitating hemolysis in this setting [9]. There are United States (US) boxed warnings for both these side effects and advice to screen all African, South-East Asian, Middle Eastern, and Mediterranean patients for G6PD prior to rasburicase administration [10]. In the following, we illustrate the morbidity and management of rasburicase-induced methemoglobinemia and hemolysis in an African-American male patient with presumed G6PD.

## Case presentation

**Figure 1:**
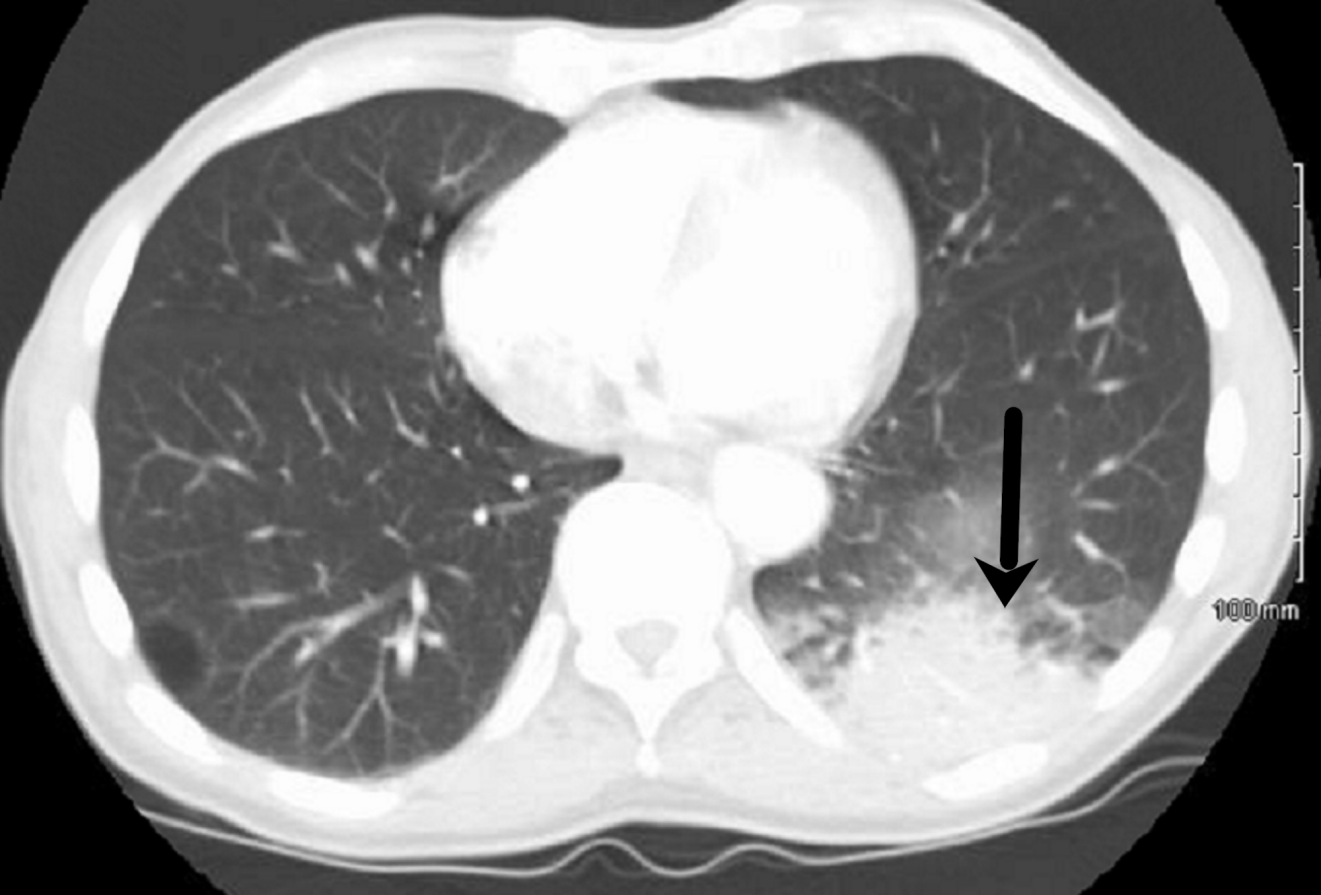
Computed tomography (CT) of the chest demonstrating a left lower lobe infiltrate

A 78-year-old African-American man weighing 53 kilograms with a past medical history significant for chronic lymphocytic leukemia (CLL), hyperuricemia with chronic gouty arthropathy, chronic kidney disease (CKD) stage 3 presented to the Emergency Department (ED) with the chief complaint of shortness of breath and fatigue for one day. His outpatient medication included ibrutinib 420 milligrams (mg) and allopurinol 300 mg daily. He was diagnosed with CLL two years prior to presentation. Initially, he was started on bendamustine with an appropriate response. However, an escalation in his lymphocyte count was appreciated one month prior to presentation and ibrutinib was initiated for CLL progression. One day prior to admission to the hospital, the patient was evaluated by his primary oncologist at an outpatient visit. He was febrile with a temperature of 101.6 degrees fahrenheit (°F) but otherwise asymptomatic. Blood and urine cultures were obtained and was started on oral levofloxacin empirically. His labs were significant for worsening hyperuricemia with a uric acid level of 13.0 milligram per deciliter (mg/dl). A single dose of intravenous rasburicase 3 mg was then administered.The following day, he presented to the ED complaining of significant fatigue associated with dry nonproductive cough of one day duration. Examination was remarkable for a fever of 102.8°F, an oxygen saturation (SPO2) of 85% on room air, conjunctival pallor and scleral icterus. A left lower lobe infiltrate was observed on his computed tomography (CT) angiogram of the chest (Figure 1). Laboratory results were noteworthy for a drop-in hemoglobin (4.9 mg/dL), indirect hyperbilirubinemia (7.2 mg/dL), low haptoglobin (<10 mg/dL) and elevated lactate dehydrogenase of 1611 International Units per liter (IU/L) compared to (756 IU/L) the day before; depicting acute hemolytic anemia. SPO2 persisted at 85% despite the use of nasal cannula, nonrebreather and non-invasive positive pressure ventilation. The patient was admitted to the intensive care unit (ICU). Arterial blood gas on 50% fraction of inspired oxygen (FiO2) was significant for an arterial oxygen level (PO2) of 222 millimeters mercury (mm Hg) and an oxyhemoglobin of 85.9%. Co-oximetry was then obtained and methemoglobin level was 13.4%. The patient was started on vancomycin and cefepime and transfused with three units of packed red blood cells. Methylene blue was not administered as the patient was assumed to have G6PD deficiency given his race and this episode of hemolysis.

After an ICU stay of two days, the patient clinically improved, SpO2 normalized, his hemoglobin levels improved, methemoglobin levels trended down and was transferred back to the floor (Table 1). A G6PD level sent during the acute attack yielded a result within the normal range. In addition, mycoplasma serum antibodies were negative. Despite broad spectrum antibiotic coverage, the patient continued to spike fevers as high as 102.7°F from day one to day four. He subsequently started complaining of right knee pain associated with right knee swelling and tenderness on examination. Arthrocentesis revealed monosodium urate crystals and was started on colchicine and prednisone. Fever, knee pain, swelling and tenderness resolved. The patient was discharged to a skilled nursing facility on prednisone taper, allopurinol and colchicine. He was continued on ibrutinib to date (five months later) and his white blood cell count is currently within normal limits. A repeat CT chest performed three monthts later documented resolution of the previously seen left lower lobe pulmonary inifiltrate.

**Table 1:** The patient’s clinical and laboratory values * Day 0 is clinic day (day prior to admission). NA: not available; g/dl: grams per deciliter; IU/L: international units per liter; mg/dl: milligrams per deciliter.

Value	Normal range	Day 0*	Day 1	Day 2	Day 3	Day 4	Day 5	Day 6	Day 7
Methemoglobin	0-1.5 %	NA	13.4	4.4	NA	1.4	NA	NA	NA
Hemoglobin	13-17 g/dl	8.3	4.9	8.0	8.1	8.6	8.2	7.9	7.9
Oxygen saturation	95-100 %	100	75-88	92-100	98-100	99-100	99-100	99-100	99-100
Lactate dehydrogenase	313-618 IU/L	756	1611	NA	2354	1713	1428	1224	1007
Total bilirubin	0.2-1.3 mg/dl	2.2	7.2	8.6	8.4	4.7	2.0	1.7	1.5
Uric acid	2.5-7.0 mg/dl	12.9	5.7	3.9	4.5	4.6	4.4	4.9	4.8

## Discussion

We present a rare case of rasburicase-induced methemoglobinemia and hemolytic anemia in the setting of G6PD deficiency. According to an international TLS expert consensus panel developed by Cairo et al., our patient was at high risk for developing TLS confirming the appropriate use of rasburicase in this scenario [11]. As previously mentioned, there is a US boxed warning advising to screen all African, South-East Asian, Middle Eastern and Mediterranean patients for G6PD prior to rasburicase administration [10]. Due to the urgency of the situation and the patient’s worsening uric acid levels, despite being on allopurinol, our patient was not screened for G6PD prior to administration. The benefits of treating this oncological emergency outweighed the risks of administering rasburicase in a patient whose G6PD status is unknown. An important aspect in testing G6PD enzymatic activity is the timing. During acute hemolysis, the RBCs with severely reduced G6PD activity will hemolyze. So, the measured assay will reflect the activity of G6PD in the remaining RBCs which carry enzymes with a relatively normal activity, resulting in a false negative result.

Methemoglobinemia occurs when the ferrous ions incorporated in hemoglobin are oxidized to ferric ions [12]. Ferric ions are unable to reversibly bind to oxygen; thus, increasing the affinity of the remaining ferrous hemoglobin to oxygen, shifting the oxygen dissociation curve to the left and therefore decreasing the release of oxygen to the tissue inducing tissue hypoxia [12]. One of the cellular reactions involved in reducing methemoglobin to hemoglobin in the body utilizes nicotinamide adenine dinucleotide phosphate (NADPH) generated by the G6PD in the hexose monophosphate shunt. This reaction is not physiologically functional and requires activation by electron carriers such as methylene blue or riboflavin [13]. Therefore, the first line of therapy for the management of methemoglobinemia, in addition to avoiding the precipitating chemical, involves the use of methylene blue to facilitate the reduction of ferric to ferrous ions [14]. In patients with G6PD deficiency, NADPH generation is reduced thus this reaction is deemed useless and methylene blue is ineffective in such patients. Furthermore, methylene blue has oxidant potential which may induce hemolysis in G6PD deficient patients [15]. In these patients, ascorbic acid is recommended [16]. Ascorbic acid is slow in onset and requires up to 24 hours and therefore is a poor choice in such emergencies [16]. Reviewing the literature, only one case was successfully managed using ascorbic acid alone [9]. Other alternative therapies include simple or exchange blood transfusions and/or hyperbaric oxygen therapy. Given the combination of hemolysis and mild methemoglobinemia <20% in our patient, we opted for a simple blood transfusion.

As per our knowledge, the number of rasburicase-induced methemoglobinemia reported cases is limited. Having both methemoglobinemia and acute hemolysis makes this case exceedingly rare. We suggest that simple transfusion alone is an effective alternative in the management of rasburicase-induced methemoglobinemia and hemolytic anemia, particularly in mild to moderate cases with methemoglobin levels less than 20%. In such scenarios, simple transfusion serves two purposes: correcting the anemia and counteracting methemoglobinemia by supplying normal hemoglobin species preventing tissue hypoxia.

(Abstract: Younis M, Eikermann SM, Hamarshi MS. Not the Typical Pneumonia; an Unusual Case of Rasburicase Induced Methomoglobinemia. American Thoracic Society conference; May 18-23, 2018). https://www.atsjournals.org/doi/abs/10.1164/ajrccm-conference.2018.197.1_MeetingAbstracts.A6921

## Conclusions

Rasburicase-induced methemoglobinemia and hemolytic anemia have been identified as a direct cause for morbidity and mortality in high-risk individuals. Methylene blue is strongly discouraged for rasburicase-induced methemoglobinemia even if the G6PD status is unknown. Management with simple blood transfusions in mild to moderate severity cases is a relatively safe and effective strategy. Spreading awareness and using order sets to remind physicians of risks associated with rasburicase and methylene blue use might be helpful. It is enouraged to report such cases and highlight the management to help set up the stage to formulate a unified consensus for treatment.
